# Leadership and Research Productivity in Japan's Academic Departments of Regional Healthcare: A Nationwide Cross‐Sectional Study

**DOI:** 10.1002/jgf2.70120

**Published:** 2026-04-22

**Authors:** Hiroki Yasuhara, Satoshi Inaba, Shinya Aoki, Takeshi Kawaguchi, Tomoki Ikai, Takashi Watari

**Affiliations:** ^1^ Division of Primary Health Care, Faculty of Medical Sciences University of Fukui Fukui Japan; ^2^ Department of General Internal Medicine Fukuchiyama City Hospital Kyoto Japan; ^3^ Department of General Medicine & Community Healthcare Kyoto Prefectural University of Medicine Kyoto Japan; ^4^ Department of General Medicine, Shioda Hospital Chiba Japan Katsuura Chiba Japan; ^5^ Department of Community‐Oriented Medical Education, Graduate School of Medicine Chiba University Chiba Japan; ^6^ Department of Emergency and Critical Care Medicine St. Marianna University School of Medicine Kawasaki Japan; ^7^ Integrated Clinical Education Center Kyoto University Hospital Kyoto Japan; ^8^ General Medicine Center Shimane University Hospital Shimane Japan

**Keywords:** general medicine, Japan, leadership, regional healthcare, research productivity, specialty distribution

## Abstract

**Background:**

Academic departments related to regional health care in Japan play a crucial role in addressing healthcare disparities and workforce shortages. However, a comprehensive understanding of their structures, leadership, and activities is lacking. This study aimed to provide the first nationwide profile of academic departments related to regional healthcare (chiiki iryou) in Japanese medical schools.

**Methods:**

A cross‐sectional study of 82 Japanese medical schools was conducted in 2024. Data on departments whose names included *chiiki* and their chairpersons were collected from official university websites and the National Physician Registration Database. To assess research output, a PubMed search was conducted for English‐language original articles published over a 2‐year period, where the chairperson was listed as the first, second, or last author.

**Results:**

A total of 145 chairpersons from 168 eligible departments were included in the analysis. Most of the departments were at national universities (66.1%), were headed by endowed chairpersons (78.0%), who were predominantly male (94.5%). Analysis of chairperson characteristics revealed: (1) a predominance of Internal Medicine specialists (44.1%) and a limited proportion of certified General Medicine specialists (10.3%); (2) limited research output, with 36.6% having no PubMed‐indexed articles published during the study period; (3) a research focus primarily on clinical (59.9% of articles) and basic science (15.6% of articles), with few articles focusing on regional healthcare issues.

**Conclusions:**

This nationwide study reveals a lack of diversity in chairperson characteristics, with a predominance of Internal Medicine specialists, limited international research output, and a possible mismatch between academic focus and regional healthcare needs.

## 1 Introduction

The importance of Community and Rural Health Care has been discussed globally. For example, in the United States, the excess mortality rate in nonmetropolitan areas has significantly increased since the 1980s [1], and the shortage of healthcare professionals in rural areas remains a major policy challenge [2, 3]. In Japan, demographic shifts influenced by the transition to a super‐aged society have occurred, and physician shortages in areas with high aging rates have been a long‐standing concern [4, 5, 6]. To address this situation, educational programs aimed at fostering and retaining medical students and junior physicians in rural areas have been promoted in various countries, including Japan [7, 8]. Notably, studies have revealed that clinical training experience in rural areas enhances physicians' motivation to work in rural areas [9, 10] and that educational and mentoring environments in regional settings influence physician retention [11]. This suggests that the development of education that considers community characteristics is important. Research on regional healthcare, which focuses on the care of residents in the community and policy interventions, is gaining attention as a field that can contribute to addressing health disparities and improving local healthcare services [12]. For example, universities in Australia actively conduct research tailored to the circumstances of specific regions, and some reports indicate that these activities directly contribute to rural health [13]. Thus, university departments play a central role in promoting educational and research activities rooted in specific regional and community contexts.

The importance of healthcare provision within specific geographical and social contexts is recognized internationally [2, 12]. However, the definitions of the target “region” or “community” vary and different terms tend to be used with relatively distinct meanings. In English‐speaking contexts, terms such as “rural” or “non‐metropolitan” are defined by population density or distance [1, 3, 13]; “underserved” indicates limited access to healthcare [4, 11]; and “community” emphasizes a population‐based approach. In contrast, the Japanese terms *chiiki* (often translated as region, area, or community) and *chiiki iryou* (regional/community healthcare) are broader and more context dependent. Depending on the context, they can encompass various aspects implied by these English terms, ranging from healthcare in remote and rural areas to primary care systems in diverse settings and health initiatives within specific communities. Reflecting this broad scope and growing needs, the number of university departments whose names include the term *chiiki* (referred to in this paper as “departments related to regional healthcare” [DRH]) has been increasing in Japan in recent years [14]. Consequently, DRH represents diverse groups with various missions. Acknowledging this Japanese context, rather than pre‐limiting the scope to specific English concepts such as “rural” or “community,” this study adopted an operational definition encompassing departments whose official Japanese names include the term *chiiki*. This approach provides an initial comprehensive overview of the diverse departments in Japan.

Despite the increasing number of DRH, their comprehensive distribution, leadership expertise, and research activities remain underexplored. The chairperson influences a department's direction, particularly in DRH where their expertise shapes responses to regional needs and research focus [15]. Although previous studies on Japanese medical institution leadership have shown gender and specialty distribution imbalances [16], the characteristics of DRH chairpersons are unknown. Understanding these aspects is crucial for regional health policy, academic development, and optimizing the contribution of DRH.

Therefore, this study aimed to investigate all DRH in Japanese medical schools to clarify their overall distribution and characteristics, with a particular focus on their chairpersons' professional backgrounds and research activities.

## 2 Methods

### 2.1 Study Design

This study employed a cross‐sectional descriptive design and included all 82 universities in Japan with medical schools as of November 1, 2024. The process involved: 1 identifying departments whose official Japanese names included “*chiiki*” (region/community) from each university's official website; 2 identifying the chairperson(s) of each department from the university website; and 3 verifying chairperson identity using Japan's National Physician Registration Database.

### 2.2 Eligibility Criteria

Departments eligible for this study included those within the 82 universities in Japan with medical schools, whose official names explicitly included the term *chiiki* and for which information was publicly available on the university's official website as of November 1, 2024. Departmental chairpersons eligible for this study were physicians listed as the chairpersons of eligible departments who could be uniquely identified through Japan's National Physician Registration Database. Chairpersons who could not be identified using the database, or who were confirmed to be nonphysicians, were excluded from the analysis of chairperson characteristics.

### 2.3 Data Collection

Data were collected regarding departmental characteristics, chairperson profiles, and specialty classifications. For each department, the following information was obtained: the official department name, the type of university (categorized as national, public, or private), and the type of department (classified as endowed, regular, or specially established, based on publicly available university records on endowed chairs). Additionally, we determined whether the department title included references to specific anatomical areas, organs, or clinical specialties.

To assess the chairpersons' profiles, data were collected on gender (as recorded in the national physician registration database), academic rank (e.g., Professor/Chief Professor, Specially Appointed Professor, Associate Professor/Lecturer), and the presence of concurrent appointments in other departments. The specialty of each chairperson was classified based on information obtained from official university websites and verified against the 19 core specialties defined by the Japanese Medical Specialty Board [17]. Additional sources included individual research achievements and biographical profiles. In cases where a chairperson could be assigned to multiple specialties, the final classification was determined through discussion among coauthors with relevant expertise. This decision was made by considering the individual's departmental affiliation, research focus, and involvement in relevant academic societies.

### 2.4 Literature Search and Research Productivity

To evaluate the research productivity of the chairpersons, a literature search was conducted of original research articles published in English. This study used PubMed as the sole data source, consistent with established practices in bibliometric analysis [18, 19, 20]. The choice of PubMed was based on three key considerations. First, it is the primary database for international publication for academic physicians in Japan, making it the most relevant source for this study's context. Second, its standardized indexing with MeSH and structured metadata ensures reliable data for bibliometric analysis. Third, as a freely accessible database, it guarantees the universal reproducibility of our findings, a critical aspect of this type of research. The search parameters included publications dated between November 1, 2022, and October 30, 2024. This 2‐year period was chosen to capture current research activity and recent academic contributions, balancing the need for up‐to‐date information with the feasibility of a nationwide survey. Each chairperson's full name was used as the author query, and the affiliation field was required to contain the official English name of the corresponding university. Eligible articles were limited to those in which the chairperson was listed as the first, second, or last author. Publications categorized as reviews, case reports, case series, guidelines, commentaries or discussions, editorials, letters, errata or corrections, and reports or conference proceedings were excluded from the analysis. Original articles were categorized into nine fields, referencing the classifications used for the Grants‐in‐Aid for Scientific Research (KAKENHI): basic science, clinical research, public health, preventive medicine, medical education, health services research, safety and quality, and other [21]. When classification was challenging, two researchers independently categorized the articles. In cases of disagreement, the final classification was determined through discussion after careful review of the article's objectives and methods. A chairperson's primary research field was defined as the KAKENHI category in which they had the highest number of publications. If publications were equally distributed across multiple fields, it was deemed “Unclassifiable.”

### 2.5 Analysis Methods

This study aimed to describe the status of the DRH and its chairpersons. Analyses were performed using a descriptive epidemiological approach. The data analyzed included department characteristics (e.g., department type, university type, and inclusion of specific names in the title), chairperson characteristics (gender, academic rank, specialty, and concurrent appointments), and classification of research productivity. Basic descriptive statistics such as frequency distributions, percentages, means, and medians were calculated to systematically describe the observed trends.

### 2.6 Ethical Considerations

This study was approved by the Ethics Review Committee of the University of Fukui (approval no. 20240138).

## 3 Results

### 3.1 Department Establishment and Characteristics

Based on the information confirmed on the official websites of all 82 universities with medical schools in Japan as of November 1, 2024, 168 departments were identified with the term *chiiki* in the name.

By university type, national universities accounted for the largest number with 111 departments (66.1%), followed by private universities with 36 departments (21.4%) and public universities with 21 departments (12.5%) (Table 1). By department type, 131 departments (78.0%) were headed by endowed chairs, 36 (21.4%) were regular departments, and 1 (0.6%) was a specially established department (Table 1).

**TABLE 1:** Characteristics of departments related to regional health care (*n* = 168).

Characteristic	Category	Number of departments	Percentage (%)
University type	National	111	66.1
	Public	21	12.5
	Private	36	21.4
Department type	Endowed chair	131	78
	Regular department	36	21.4
	Other (specially established)	1	0.6
Inclusion of specific area name in title	Included	19	11.3
	Not included	149	88.7
Inclusion of specific organ/disease/specialty name in title	Included	73	43.5
	Not included	95	56.5

An analysis of department names revealed that only 19 departments (11.3%) included specific area names in their titles, whereas the majority (149 departments, 88.7%) did not (Table 1). Furthermore, 73 departments (43.5%) had names that included specific organs, diseases, or specialties, whereas 95 departments (56.5%) did not (Table 1).

### 3.2 Chairperson Characteristics

Among the 168 identified departments, 145 listed chairpersons were uniquely identified through the national physician registration database or similar sources and were included in the analysis of chairperson characteristics. Sixteen individuals were excluded: 14 because their identity as chairpersons could not be confirmed from publicly available information, one because multiple physicians with the same name were included in the physician database, preventing unique identification, and one because they were confirmed to be a nonphysician. Of the 145 chairpersons, eight held concurrent appointments across more than one DRH (with one individual chairing three DRH), and two departments had two co‐chairpersons. These factors accounted for the discrepancy between the number of departments and the number of chairpersons and resulted in 145 unique chairpersons for analysis.

Of the 145 chairpersons, 137 (94.5%) were male and 8 (5.5%) were female, indicating a male predominance (Table 2). The most common academic rank was “Professor” or “Chief Professor” (88 chairpersons, 60.7%), followed by “Other Professor” (e.g., Specially Appointed Professor or Designated Professor) (31 chairpersons, 21.4%), and “Other” (e.g., Associate Professor or Lecturer) (26 chairpersons, 17.9%) (Table 2).

**TABLE 2:** Characteristics of chairpersons of departments related to regional healthcare (*n* = 145).

Characteristic	Category	Number (n)	Percentage (%)
Gender	Male	137	94.5
	Female	8	5.5
Academic rank	Professor or Chief Professor	88	60.7
	Professor (Other ranks/titles)^a^	31	21.4
	Other (e.g., Assoc. Prof., Lecturer)^b^	26	17.9
Concurrent appointments	Yes	57	39.3
	No	88	60.7
Years since obtaining medical license	Mean (years)	32.8	—
	Median (years)	33	—
	Minimum (years)	16	—
	Maximum (years)	54	—

^a^ Includes ranks such as Specially Appointed Professor and Designated Professor.

^b^ Includes ranks such as Associate Professor and Lecturer.

In 2024, at the time of the survey, the average number of years since obtaining a medical license was 32.8 years, with a median of 33.0 years (range: 16.0–54.0 years) (Table 2). Of the 145 chairpersons, 88 (60.7%) held no concurrent positions in other departments or divisions, whereas 57 (39.3%) held at least one concurrent appointment (Table 2).

### 3.3 Chairperson Specialty and Research Activities

**FIGURE 1:**
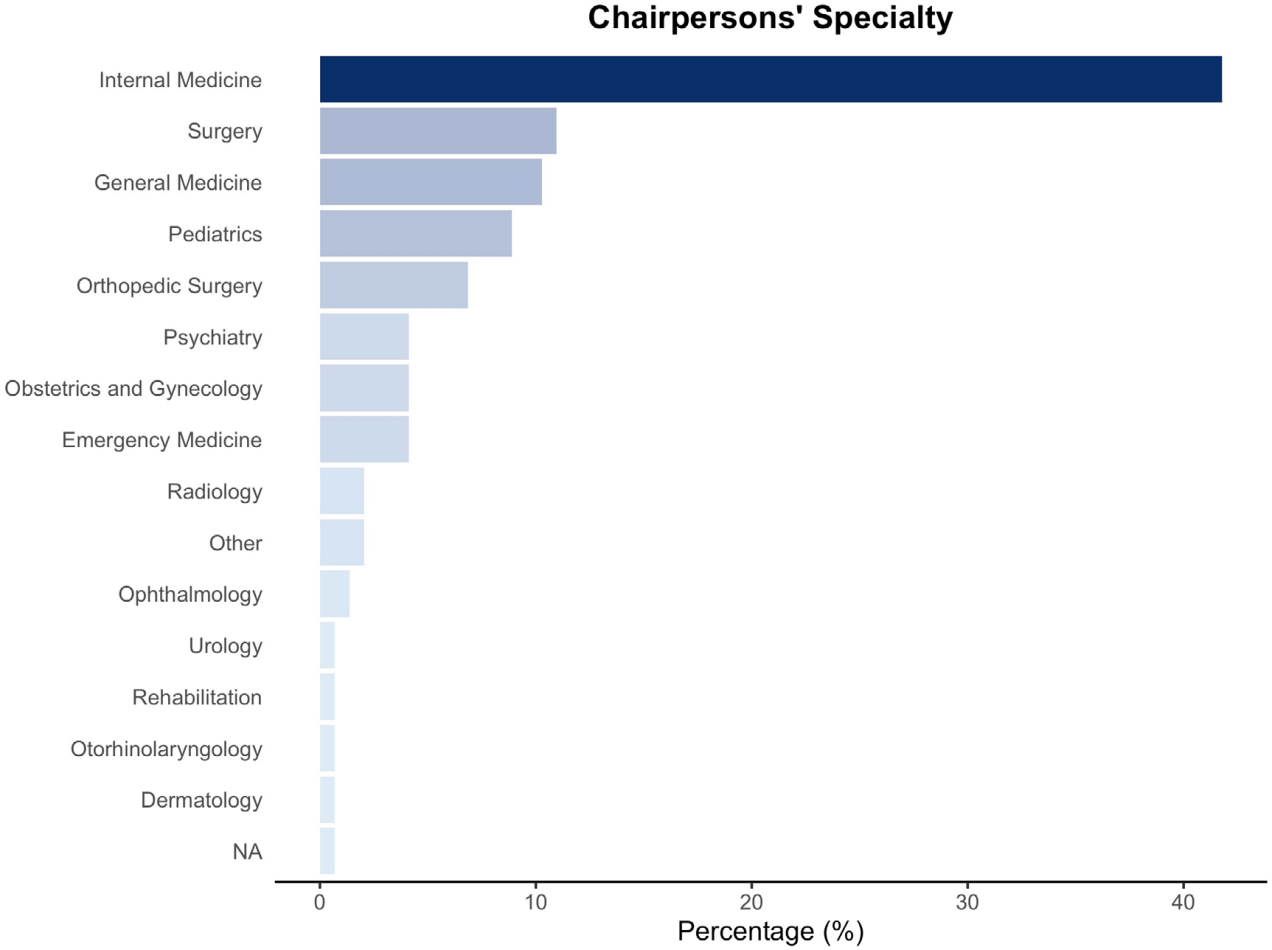
Chairpersons' specialty.

The chairpersons' primary clinical specialties (based on 19 fundamental specialty areas defined by the Japanese Medical Specialty Board) varied widely. Internal Medicine was the most common specialty (64 chairpersons; 44.1%), followed by Surgery (16 chairpersons; 11.0%), General Medicine (15; 10.3%), Pediatrics (13; 8.9%), Orthopedic Surgery (10; 6.8%) (Figure 1). No DRH chairpersons had specialized in Neurosurgery, Pathology, Clinical Laboratory Medicine, Plastic Surgery, or Anesthesiology.

The PubMed search identified a total of 799 original articles published by the 145 chairpersons during the 2‐year study period (November 1, 2022, to October 30, 2024) in which the chairperson was the first, second, or last author. (The total number of articles included 10 articles counted twice owing to involving two eligible chairpersons as authors.) Classification of these articles by research field showed that clinical research was the most frequent category (479 articles; 59.9%), followed by basic science (125 articles; 15.6%) and public health (90 articles; 11.3%). Other fields included medical education (43 articles; 5.4%), safety and quality (26 articles; 3.3%), health services research (25 articles; 3.1%), and preventive medicine (2 articles; 0.2%). Nine articles (1.1%) could not be classified (Table 3).

**TABLE 3:** Distribution of chairpersons' primary research fields and published articles by field.

Research field	Chairpersons by primary field (*n* = 145)	Published articles (*n* = 799)	Published articles (%)
Basic science	13	125	15.6
Clinical research	51	479	59.9
Public health	11	90	11.3
Preventive medicine	0	2	0.3
Medical education	7	43	5.4
Health services research	0	25	3.1
Safety and quality	1	26	3.3
Unclassifiable^a^	9	9	1.1
No publications during period	53	—	—
Total	145	799	100

^a^ Primary research field could not be determined due to an equal number of publications across multiple fields (tie).

**FIGURE 2:**
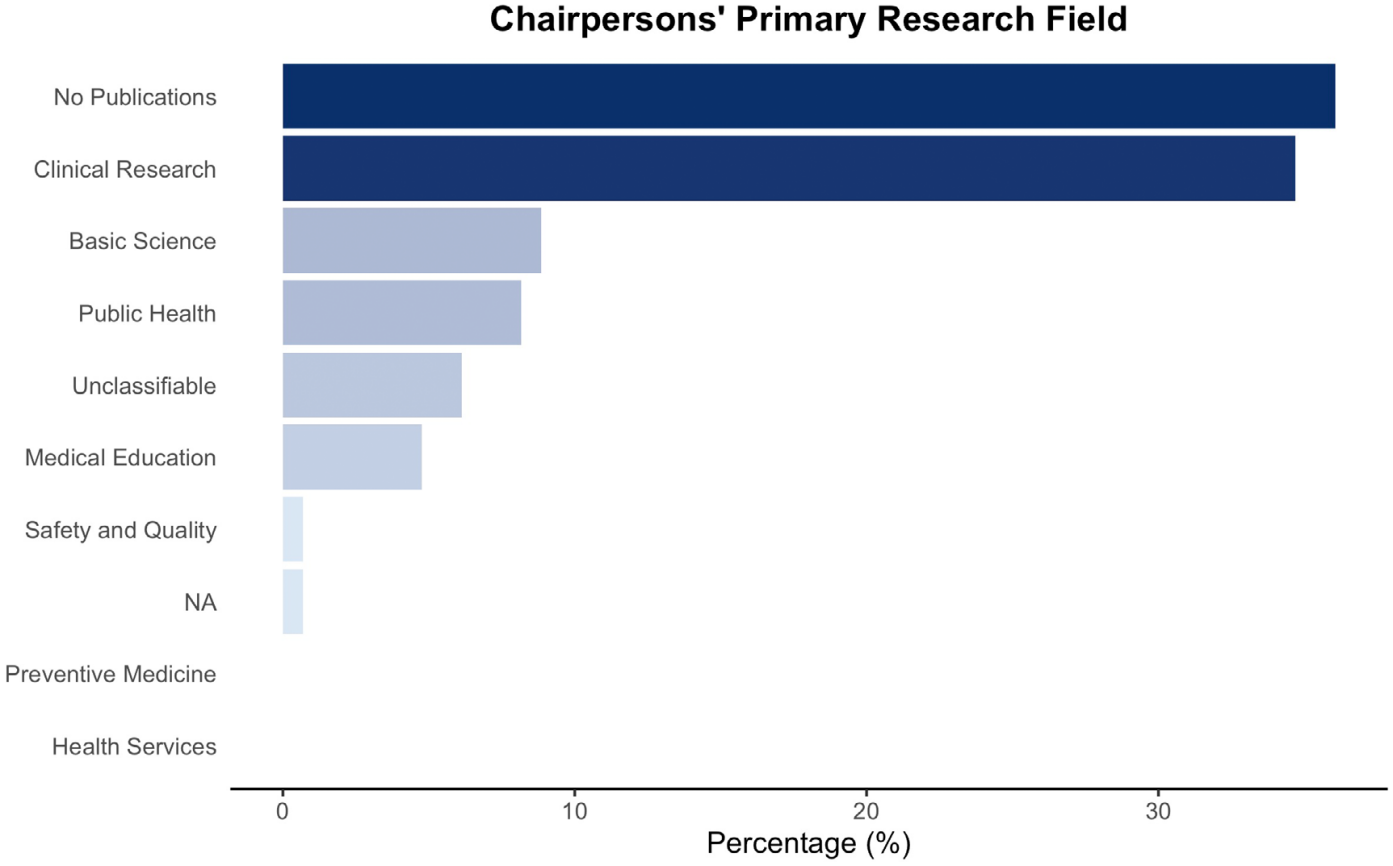
Chairpersons' primary research fields.

Among the 145 chairpersons, clinical research was the most common primary field for 51 chairpersons (35.2%), followed by basic science (13 chairpersons, 9.0%), public health (11 chairpersons, 7.6%), and medical education (7 chairpersons, 4.8%), and one chair (0.7%) focused primarily on safety and quality. None of the chairpersons had preventive medicine or health‐service research as their primary field, and the primary research field of 9 chairpersons (6.2%) could not be classified (Table 3 and Figure 2). Notably, during the study period, 53 chairpersons (36.6%) had no eligible publications identified in PubMed (Table 3). Of the 13 chairpersons whose primary research field was basic science, 12 (92.3%) held concurrent appointments in other departments or divisions.

## 4 Discussion

To the best of our knowledge, this study is the first detailed investigation of DRH, known as c*hiiki* in Japan. It examined their distribution and characteristics, as well as chairpersons' characteristics, specialties, and research activities. The findings reveal that most chairpersons were physicians specialized in Internal Medicine, with only a minority holding certifications in General Medicine. This discrepancy is not surprising given the large number of Internal Medicine specialists relative to General Medicine specialists in Japan: In April 2025, 44,069 physicians were board‐certified in Internal Medicine, whereas only 937 were board‐certified in General Medicine, highlighting a substantial difference in availability [22, 23]. Furthermore, approximately one‐third of the chairpersons had no identifiable original English language publications indexed in PubMed during the 2‐year study period. Although clinical research was the primary focus of chairpersons' publications, a sizable minority had basic scientific research as their primary research focus.

The limited research output among some chairpersons is similar to the findings of Tago et al. [24] regarding General Medicine departments in Japanese universities. Although this study did not directly assess workload or support systems, it is plausible that heavy commitments in education, clinical practice, and community engagement, alongside potentially insufficient research infrastructure, contribute to these challenges, as suggested by the research productivity model proposed by Bland et al. [25] provides a useful framework for a multifaceted understanding of the challenges to research productivity observed in this study. According to this model, individual characteristics (e.g., research motivation and skills), organizational characteristics (e.g., research support systems and departmental culture), and leadership characteristics (e.g., research promotion ability) tend to be interrelated. Although this study was unable to identify causal relationships between these factors owing to the cross‐sectional study design, it underscores the need for further research to clarify which of these elements pose challenges to research productivity and how they interact.

Second, although clinical research was the predominant primary research field for chairpersons in this study, the finding that 9.0% of chairpersons focused primarily on basic science and that basic science constituted 15.6% of all publications warrants discussion. A survey of General Medicine departments in Japanese university hospitals by Watari et al. [26], showed that although clinical research was the primary focus, basic science research accounted for 9% of research output, similar to our findings. However, an analysis of publications from Australian University Departments of Rural Health by Gausia et al. [13] found that more than half (56%) of the publications were related to rural health issues. Compared with this Australian example, the research fields in this study differ markedly from those of DRH at Australian universities, even if the 43.5% of departments with specific organ/disease/specialty names in their titles are excluded, revealing that *chiiki* departments in Japanese medical schools may not always be directly linked to the clinical and public health healthcare needs of their specific location. Morris et al. [27] noted that the time lag for basic science to translate into clinical practice is generally substantial, often cited as an average of 17 years. This lengthy period makes it difficult for basic science research to respond rapidly to pressing regional healthcare challenges. However, basic science discoveries are fundamental for future medical advancements and can offer novel approaches to local health problems. The challenge lies in optimizing translational pathways and aligning basic research with long‐term regional health goals. Therefore, these results do not necessarily indicate a disconnect of research focus from community needs. Further investigation, such as mapping research themes to specific regional health challenges, is required to assess the alignment more thoroughly. The finding that many chairpersons whose primary research field was basic science had concurrent appointments suggests a potential link between the chairperson's professional background, structure of concurrent appointments, and selection of research fields.

Third, as pointed out by Takahashi et al. [28], the small number of chairpersons specializing in General Medicine reflects the current situation in Japan, such as the short history and small absolute number of the Japanese General Medicine specialist system. Although a 2019 survey indicated that 90.5% of responding universities had General Medicine departments, many of which run specialist programs [29], our findings suggest that only a small minority of DRH have General Medicine specialists in leadership roles. This highlights a need to consider the role of General Medicine expertise in DRH leadership, given their mission of contributing to regional health care. It is unsurprising that Internal Medicine specialists, who have historically close ties with local health care and abundant human resources, tend to assume leadership roles in DRH given the shortage of General Medicine specialists in Japan.

Furthermore, interpreting the research activities of DRH chairpersons requires considering their substantial educational responsibilities, particularly within Japan's regional quota program. The program, designed to address physician maldistribution, makes it mandatory for graduates receiving scholarships to work in specific regions [30]. DRH faculty, especially chairpersons, are heavily involved in educating students about this system, providing career guidance, and coordinating with local stakeholders. These extensive duties may constrain resources available for research. Thus, evaluating DRH leadership necessitates a multifaceted perspective, considering both research outputs and educational contributions to fostering regional healthcare physicians.

This study has several strengths. First, it covered all medical schools nationwide, providing the first comprehensive overview of DRH in Japan, which was previously unclear. Second, data were collected and analyzed from multiple perspectives, encompassing aspects such as department distribution and characteristics, and chairperson attributes, specialties, and research activities. Third, in order to be as objective as possible, we collected information based on publicly available information sources, including official university websites, physician registration data, and PubMed. These findings provide essential foundational information to facilitate evidence‐based discussion regarding future strategies for developing human resources for community and rural healthcare, management of DRH, and national‐level policymaking.

This study also has some limitations. First, using “*chiiki*” for department selection might have introduced selection bias, as functionally similar departments with different names may have been missed, and the departments included in this study may have had differing missions. Chairperson identification via websites may not always reflect actual leadership or capture multiple leaders. Second, our bibliometric analysis relied solely on PubMed. As detailed in the Methods section, this approach was intentionally chosen to align with the specific context of our study and to prioritize data consistency and reproducibility. However, this decision presents a clear limitation. Our study does not capture publications indexed in other major international databases (e.g., EMBASE, Scopus), nonbiomedical databases (e., ERIC), or domestic databases (e., Ichu‐shi). This methodological choice represents a trade‐off between a focused, reproducible analysis and comprehensive coverage. Consequently, our findings likely underestimate the full extent of the chairpersons' scholarly output, and future studies employing a multidatabase search strategy would be valuable in providing a more comprehensive assessment. For example, although Web of Science has long been regarded as one of the most comprehensive citation databases worldwide, PubMed offers more granular bibliographic information, including publication types such as scientific commentaries [31]. Combining these distinct strengths would enable a more multifaceted evaluation of academic productivity. Third, the KAKENHI‐based research field classification might not fully capture interdisciplinary regional healthcare research. Finally, the cross‐sectional design precludes establishing causality or observing changes over time. Future research should investigate factors influencing research productivity, explore support measures, evaluate research alignment with community needs, and analyze the impact of chairperson leadership on departmental outcomes (education, research, and community contributions).

## 5 Conclusion

This investigation of university departments in Japan whose names include *chiiki* identified a lack of diversity in chairperson specialties, with a predominance of Internal Medicine specialists and few holding certification in General Medicine, which focuses on local healthcare. Although this probably reflects the historical context and the absolute number of specialists in each field in Japan, the leadership composition of DRH warrants further discussion to ensure that it is optimally aligned with the diverse, interdisciplinary, and community‐focused missions of these departments. Furthermore, in this study, approximately one‐third of the chairpersons had no identifiable original English‐language publications indexed in PubMed during the 2‐year study period, indicating possible challenges in research productivity. Published research has primarily focused on clinical and basic science, with a limited number of publications focusing on regional healthcare. These findings provide an important foundation for future discussions on strengthening the functions of DRH and developing human resources for regional healthcare provision. Addressing these challenges could strengthen DRH in Japan, thereby enhancing their contribution to building a sustainable community and rural healthcare system and improving the health of community residents.

## Author Contributions

**Hiroki Yasuhara:** conceptualization, methodology, software, data curation, investigation, validation, formal analysis, resources, visualization, writing – original draft, writing – review and editing. **Shinya Aoki:** conceptualization, methodology, data curation, validation, formal analysis, visualization, writing – original draft, writing – review and editing. **Satoshi Inaba:** conceptualization, methodology, software, formal analysis, data curation, resources, validation, visualization, investigation, writing – original draft, writing – review and editing. **Tomoki Ikai:** writing – original draft, supervision. **Takeshi Kawaguchi:** conceptualization, methodology, formal analysis, visualization, writing – original draft, writing – review and editing. **Takashi Watari:** conceptualization, methodology, software, data curation, supervision, formal analysis, validation, visualization, writing – review and editing, resources, writing – original draft, funding acquisition, investigation, project administration.

## Funding

The authors have nothing to report.

## Conflicts of Interest

The authors declare no conflicts of interest.

## Data Availability

The data that support the findings of this study are available on request from the corresponding author. The data are not publicly available due to privacy or ethical restrictions.
